# Influence of internet usage on physical activity participation among Chinese residents: evidence from 2017 China General Social Survey

**DOI:** 10.3389/fpubh.2024.1293698

**Published:** 2024-05-30

**Authors:** Min Ji, Detian Deng, Xiaojuan Yang

**Affiliations:** ^1^School of Political Science and Public Administration, Henan Normal University, Xinxiang, China; ^2^Department of Social and Historical Sciences, Technical University of Darmstadt, Darmstadt, Germany

**Keywords:** Chinese context, China General Social Survey, internet usage, physical activity, moderating effects

## Abstract

**Objectives:**

This study aimed to examine the impact of internet usage on physical activity participation among Chinese residents, utilizing data from the 2017 China General Social Survey (*N* = 12,264). The objectives were to investigate the relationship between internet usage and physical activity participation and to explore the moderating effects of gender, age, and education level.

**Methods:**

Multiple regression models and a binary Probit model were employed to analyze the data. The study focused on exploring the association between internet usage and physical activity participation, considering the moderating effects of gender, age, and education level. The sample consisted of 12,264 participants from the 2017 China General Social Survey.

**Results:**

The study found a positive association between increased internet usage and decreased engagement in physical activity, suggesting a negative influence of internet usage on physical activity. Significant age-related moderating effects were observed, indicating varying patterns of the internet-physical activity relationship across different age groups. Gender and education level were also found to significantly moderate this association, highlighting the impact of gender equality and educational attainment on individuals' utilization of the internet for physical activity purposes.

**Conclusion:**

This study underscores the evolving role of the internet in shaping physical activity behaviors in the Chinese context. It emphasizes the importance of considering age-related dynamics and societal factors such as gender equality and educational attainment in health promotion strategies.

## Introduction

The proliferation of the internet has transformed Chinese lifestyles, profoundly impacting communication patterns, information accessibility, and leisure activities (1, 2). The internet has become a pervasive force that shapes the way individuals think, exchange ideas, and engage in various behaviors. This transformation is evident in the widespread use of digital platforms for communication and the integration of online activities into daily routines. In the field of physical activities, the internet has become a significant social phenomenon and economic resource (3–5). It provides new platforms and development opportunities for physical activities. With the popularization and application of the Internet, physical activities can not only spread information through newspapers, broadcasts, and other means, but also make it easier for more people to obtain registration for physical activities and sports training courses through the Internet. This provides new opportunities for improving the dissemination and participation of physical activities. In the digital age, the internet has greatly enriched people's lives, expanded their range of activities, and enhanced their abilities and levels of engagement in physical activities. The internet plays a pivotal role in enhancing residents' abilities and levels of engagement in physical activities. Through online platforms, residents can access a wealth of information about physical activities, including details about sports facilities such as locations, opening hours, and registration methods. This accessibility significantly facilitates residents' participation in various physical activities.

Moreover, the internet provides residents with the opportunity to engage in online training and guidance resources, accessible anytime and anywhere, enabling them to participate in physical activities under professional guidance. For instance, individuals can access virtual fitness classes, personalized workout routines, and expert-led training sessions through online platforms. Furthermore, the Internet also promotes communication and interaction among residents, by participating in online discussions and social media groups to share experiences and establish connections. This interconnectedness contributes to a sense of community, encouraging residents to stay motivated and engaged in their physical activity pursuits. The Internet has become an indispensable medium for physical activities participation (6, 7).

Physical activities are unique social activities that involve three dimensions: the interaction between individuals and nature, individuals and society (8, 9). Physical activities, as a special form of social activity, encompass various activities aimed at enhancing and regulating physical functions for specific purposes (10, 11). Depending on the goals, physical activities can be categorized into medical exercise, fitness activities, and recreational activities. Based on the participants, physical activities can be divided into individual participation and collective participation (12, 13). In the Internet era, digital technology provides a massive platform for information exchange, facilitating communication, expanding the scope of information dissemination, improving information acquisition efficiency, and enhancing residents' health conditions (7, 14).

Research has shown that the internet plays three key roles in promoting citizens' engagement in physical activities. Firstly, the internet serves as a catalyst for increased physical activity participation. With the rapid development of Internet technology and the advent of the digital age, the physical activity participation rate among Chinese citizens has significantly increased (4, 15). According to the 2020 Survey Report on the Status of National Fitness Activities released by the National Physical Fitness Monitoring Center (16), the proportion of residents aged 7 and above who participated in physical exercise once or more per week in 2020 was 67.5%, with 70.4% in urban areas and 63.1% in rural areas. Notably, when compared to the 2014 survey, the proportion of people participating in physical exercise once a week or more exhibited a substantial increase of 18.5%. Secondly, the internet functions as a versatile platform, offering avenues for entertainment, social interaction, and sport-related consumption. This multifaceted role enriches individuals' lives and contributes to a holistic engagement in physical activities. Lastly, the internet's entertainment and consumption functions collectively impact residents' physical activity participation by influencing their preferences and choices (17–19).

Previous studies have pointed out that the participation of Chinese residents in physical activities is mainly influenced by two factors: their own health conditions and participation demands (20). According to the 2023 Statistical Reports on Internet Development in China, as of June 2023, the number of Internet users in China reached 1.079 billion, with an internet penetration rate of 76.4% (21). Various internet applications continued to develop, and the number of internet users accessing the internet through mobile phones reached one billion. Self-health conditions directly affect individuals' ability to engage in physical or other activities, while participation demands are based on physical exercise and leisure entertainment considerations. Analyzing the impact of the Internet on residents' physical activities participation from these two perspectives, the following results are observed: first, Internet usage has positive relation to physical health conditions. Residents with poorer physical health (including chronic illnesses) are more likely to engage in daily sports exercises through the Internet. Second, Internet usage is positively associated with social interaction demands. Third, Internet usage has a positive effect on entertainment demands (22–24).

Overall, the Internet is an important medium and cultural phenomenon in the social life of Chinese citizens (19, 25). It provides a crucial platform and technological support for the development of citizens' physical activities. Internet usage expands people's living space and social interactions, facilitating social communication (8, 15). Through online information exchange, it meets people's information needs. It also enables understanding of consumption behavior and health conditions, facilitating health management. The Internet has brought about numerous influences: it broadens the choices for people to participate in physical activities, enhances their ability to engage in physical activities, and strengthens the motivation of residents to participate in physical activities (26, 27). Analyzing the potential impact of Internet usage on citizens' participation in physical activities can provide new ideas, methods, and approaches for promoting nationwide fitness in China. Based on the above analysis, this study primarily focuses on two questions using the 2017 China General Social Survey data:

(1) Does the internet usage have a negative impact on residents' participation in physical activities in China?
(2) From a structural perspective, do factors such as gender, education level, and income moderate the relationship between internet usage in the context of physical activity and residents' participation in physical activities?

## Literature review, theoretical analysis and hypothesis

The widespread and rapid development of the internet has had profound impacts on various sectors of society, including the field of physical activities. The influence of internet usage on physical activities participation has become a highly discussed research topic. Previous research has explored the relationship between internet usage and physical activity participation, providing valuable insights into the potential impact of internet usage on physical activities participation (28, 29). Studies have found that increased internet usage, particularly engaging in sedentary activities such as browsing social media, playing video games, or watching online content, is associated with a decrease in physical activity levels. For example, Lin and Lachman's study (30) revealed the positive role of internet usage in promoting residents' engagement in physical activities. They emphasized that the internet, as a crucial tool for information acquisition and communication, can help individuals overcome challenges related to exercise and information access, thereby stimulating interest in physical activities participation. Çetin and Kökalan (1), from the perspective of demographic variables, analyzed the impact of internet usage on residents' physical activities participation, providing a differentiated perspective for research. These studies have laid the foundation for understanding the mechanisms by which internet usage affects physical activities participation. Research by Escobar-Viera et al. (31) showed that higher screen time, which includes internet usage, was negatively associated with physical activities participation among adolescents. Similarly, a study by Wang et al. (32) found that excessive internet usage was linked to decreased engagement in physical activities among college students. Excessive internet use, characterized by disproportionate or inadequately regulated preoccupations, urges, or behaviors related to internet access resulting in impairment or distress, shares conceptual similarities with internet addiction (33). These findings suggest a potential negative relationship between internet usage and physical activities participation.

From a theoretical standpoint, social cognitive theory can partially explain the relationship between internet usage and physical activities participation (34). Social cognitive theory emphasizes that individuals acquire new knowledge and skills through observing and imitating the behaviors of others (35). In the era of the internet, people can gain rich sports-related information and knowledge by watching live sports broadcasts, sharing exercise experiences, and participating in online sports communities, thereby promoting physical activities participation. However, social cognitive theory also points out that individuals are influenced by the social environment and the behaviors of others when making choices. Thus, the content of internet information and the construction of social networks can have positive or negative effects on physical activities participation.

Although research suggests a negative correlation between excessive internet usage and the frequency of physical exercise, studies such as Vries et al. (36) have indicated the detrimental impact of internet usage on physical exercise. However, there is a need for deeper exploration of the relationship between physical activities participation among Chinese residents and internet usage, especially when considering different moderating variables.

In terms of gender, existing research has indicated differences in physical activities participation between males and females. Compared to the female population, men exhibit a more positive attitude toward physical activities (37). Males might be more likely to seek sports-related information and engage in social activities online, thereby mitigating the negative impact of internet usage on physical exercise (28, 38, 39). Conversely, females might spend more time on virtual social interactions and entertainment, potentially reducing opportunities for physical activity (14). Thus, gender might play a moderating role in the relationship between internet usage and physical activities participation.

Age and education level are also important moderating variables that may influence the relationship between internet usage and physical activities participation (3, 24, 40). Different age groups may exhibit varying attitudes and behaviors toward internet usage and physical exercise (41, 42). Younger individuals might be more attracted to internet entertainment, while older individuals might prioritize health and physical activity (28, 43). Education level can impact individuals' cognition and behavior regarding the relationship between internet usage and physical exercise (39). Those with higher education levels may be more aware of the negative health effects of internet usage, leading to more proactive physical activity participation.

Building upon the aforementioned literature review and theoretical analysis, this study proposes the following hypotheses:

*H1: Internet usage has a negative effect on physical activity participation among Chinese residents*.
*H2: Gender plays a moderating role in the relationship between internet usage and physical activity participation among Chinese residents*.
*H3: Age plays a moderating role in the impact of internet usage on physical activity participation among Chinese residents*.
*H4: Education level plays a moderating role in the relationship between internet usage and physical activity participation among Chinese residents*.

### Methodology

The study utilizes data from the 2017 China General Social Survey (CGSS2017), a comprehensive survey designed to capture diverse social and demographic aspects of the Chinese population. CGSS2017 aims to provide valuable insights into the social dynamics, attitudes, and behaviors of Chinese residents. Multiple linear regression models were employed, which is most suitable for delving into the relationship between individuals' internet usage and their participation in physical activities. In constructing the multiple regression models, we comprehensively consider various factors such as the duration and frequency of internet usage, as well as the frequency and duration of physical exercise, to examine and validate the relationship between internet usage and physical activities participation, thus confirming the H1 hypothesis. Additionally, structural factors such as gender, education level, and income are introduced as moderating variables to investigate their potential moderating effects on the relationship between internet usage and physical activity participation, validating the H2, H3, and H4 hypotheses.

Specifically, we establish a series of regression models where the dependent variable is the physical activities participation behavior, and the independent variables include internet usage duration, internet usage frequency, as well as factors like gender, education level, and income. Furthermore, in Model 2, an interaction term of internet usage and gender is introduced; in Model 3, an interaction term of internet usage and age is included; and in Model 4, an interaction term of internet usage and education level is incorporated. To control for potential confounding factors, control variables such as gender, age, education level, income, urban/rural residence, and health condition are introduced.

To ensure the robustness of the regression results, a variable substitution model is employed for analysis. The exercise frequency is categorized into five levels: daily, several times a week, several times a month, several times a year, and never. A binary Probit model is utilized for robustness test.

### Data source and variable selection

The data utilized in this study originates from the 2017 China General Social Survey (CGSS2017). analyzed with Stata 17.0. The 2017 CGSS survey employed a rigorous multistage stratified random sampling method from 28 provinces, municipalities, and autonomous regions across the country. Conducted from August to November 2017, this survey aimed to collect and organize information related to China's societal basic conditions, structural changes, and socio-economic development. It represents a large-scale, interdisciplinary comprehensive research effort. CGSS2017 data is renowned for its comprehensive, authentic, and objective characteristics. During the data collection process, apart from effective sampling, sample selection and adjustments were carried out through data organization and analysis to ensure data integrity. In the sampling process, CGSS2017 established contact with respondents through telephone communication, followed by on-site visits and information collection. This study selected CGSS2017 data as its research focus, with an effective sample size of 12,264, including 5,788 male and 6,476 female participants. The questionnaire survey encompassed individual basic information, lifestyle, physical activities participation behaviors, and intentions.

### Core explanatory variable: internet usage

The internet has become an integral part of life, profoundly altering people's lifestyles and work patterns, and it is also one of the significant variables influencing physical activities participation. This study begins with the variable of internet usage to reflect the extent of residents' internet usage in the form of a virtual variable. With reference to similar studies categorizing media types, traditional media such as newspapers, television, and radio are referred to as conventional media, while the internet, mobile phones, and computers are termed as online media due to their characteristics in media information dissemination. Drawing inspiration from the categorization of media types in similar studies, this research measures internet media usage through the question, “In the past year, how often have you used the internet (including mobile phones)?” with response options ranging from 1 (“Never”) to 5 (“Always”), where higher values indicate more frequent internet media usage. In this study, participants who selected “Never” (coded as 1) indicated non-frequent internet use, while those who selected any other option (ranging from 2 to 5) were categorized as frequent internet users (coded as 0).

### Explained variable: physical activities participation

According to previous studies (44, 45), physical activity refers to moderate to vigorous physical activity (MVPA) during a typical week and in the previous week. In the context of this study, physical activity is specifically defined as the frequency of engaging in physical activity during leisure time over the past year. In the CGSS2017 survey questionnaire, respondents were asked about their frequency of participating in physical exercise during leisure time over the past year (see CGSS2017 Questionnaire: A30-9). Response options ranged from 1 (“Every day”) to 5 (“Never”). Regarding the quantification of physical activity frequency (see CGSS2017 Questionnaire: A15a), residents who engaged in physical activities for more than 30 min per month and sweated were coded as 1, while those who did not meet this criterion were coded as 0.

### Control variables

In line with previous studies and the research hypotheses, this study considers individual variables such as gender, age, education level, income level, residence, social media usage, other internet application usage, individual social connections, individual media usage motives, social background, and personal characteristics as possible factors that might influence the relationship.

The multiple regression models are constructed as follows in Equations 1–4:


(1) $$\begin{array}{l} {\text{y}}_{\text{i}}=\beta 0+{\beta 1\text{internet}}_{\text{i}}\;+\sum \text{control}+\varepsilon_{\text{i}} \end{array}$$

(2) $$\begin{array}{l} {\text{y}}_{\text{i}}=\beta 0+{\beta 1\text{internet}}_{\text{i}}\;+{\beta 2\text{internet\_gender}}_{\text{i}}\;+\sum \text{control}+\varepsilon_{i}\; \end{array}$$

(3) $$\begin{array}{l} {\text{y}}_{\text{i}}=\beta 0+{\beta 1\text{internet}}_{\text{i}}\;+{\beta 3\text{internet\_age}}_{\text{i}}\;+\sum \text{control}+\varepsilon_{i} \end{array}$$

(4) $$\begin{array}{l} {\text{y}}_{\text{i}}=\beta 0+{\beta 1\text{internet}}_{\text{i}}\;+{\beta 4\text{internet\_edu}}_{\text{i}}+\sum \text{control}+\varepsilon_{i} \end{array}$$

Where *i* represents the *i* th sample, β0 is the intercept, β1 is the coefficient for the core explanatory variable, which is the internet usage time, β2 is the coefficient for the interaction term between internet usage and gender in Model 2, β3 is the coefficient for the interaction term between internet usage and age in Model 3, β4 is the coefficient for the interaction term between internet usage and education level in Model 4. The term “*Control*” encompasses control variables such as gender, age, education level, income, urban/rural residence, and health status. ε represents the error term.

## Results

### Descriptive statistical analysis

Table 1 presents the descriptive statistics. The logarithmic mean of weekly exercise time is 0.491, with a maximum value of 1 and a standard deviation of 0.5, which is greater than the mean, indicating significant variation in the distribution of physical exercise frequency within the study sample. The average internet usage is 0.27 with a standard deviation of 0.444, substantially exceeding the mean, suggesting considerable diversity in internet usage frequency among the study participants. The logarithmic mean of gender is 0.472, with males constituting 47.2% of the research sample. The average age is 50.782, ranging from 18 to 103. The average educational level is 1.861, corresponding to a high school education. The logarithmic mean of income is 11.161, with a maximum value of 16.118. Urban residents account for 64.5% of the sample. The average health level is 3.478, indicating the overall health status of the participants in the study.

**Table 1:** Descriptive statistics of variables (*N* = 12,264).

**Variables**	**Samples**	**Mean**	**SD**	**Min**.	**Max**.
Physical activity participation	12,264	0.491	0.500	0.000	1.000
Internet	12,264	0.270	0.444	0.000	1.000
Gender	12,264	0.472	0.499	0.000	1.000
Age	12,264	50.782	16.813	18.000	103.000
Edu	12,264	1.861	0.721	1.000	3.000
Income	12,264	11.161	2.006	1.946	16.118
Urban	12,264	0.645	0.478	0.000	1.000
Health	12,264	3.478	1.094	1.000	5.000

### Correlation analysis

The relationships between variables were examined using the Pearson correlation coefficient. The correlation analysis aimed to assess the strength and direction of relationships between physical exercise frequency (y) and other relevant variables, including internet usage, age, education level (edu), household income, and health level. Through correlation matrix analysis in Table 2, it was found that the frequency of physical activity is positively correlated with internet usage, educational level, household income, and health level. All these correlations were statistically significant at the 1% confidence level, suggesting a discernible positive trend in these factors relative to physical exercise frequency during the study period. In essence, there was a clear concurrent increase in these factors and physical exercise frequency. Conversely, the correlation between age and exercise frequency was not significantly evident, indicating the absence of a pronounced positive or negative trend. Most of the correlation coefficients among the control variables were below 0.3, suggesting the absence of severe multicollinearity issues between variables.

**Table 2:** Correlation matrix of variables.

	***y***	**Internet**	**Gender**	**Age**	**Edu**	**Income**	**Urban**	**Health**
Physical activity participation	1.000							
Internet	0.158^***^	1.000						
Age	−0.114^***^	−0.463^***^	0.010	1.000				
Edu	0.248^***^	0.456^***^	0.102^***^	−0.458^***^	1.000			
Income	0.113^***^	0.212^***^	−0.022^**^	−0.172^***^	0.286^***^	1.000		
Health	0.174^***^	0.276^***^	0.059^***^	−0.397^***^	0.334^***^	0.183^***^	0.184^***^	1.000

^**^p < 0.05.

^***^p < 0.01.

In the context of exercise frequency as shown in Table 2, the correlation with age is negative (−0.114, *p* < 0.01), indicating a significant downward trend where older individuals tend to engage in physical activities less frequently. This observed negative correlation may be attributed to factors such as physical discomfort, declining physical capabilities, or increased life pressures that potentially lead older individuals to reduce their participation in physical exercise.

### Multiple regression analysis

As indicated in Table 3, this study established four regression models to examine the impact of internet media usage on residents' participation in physical activities. In Model 1, we investigated the influence of internet usage time (internet_i) on residents' participation in physical activities (*y*_i_). In Model 2, we introduced gender (internet_gender_i) as a moderating variable to explore its potential impact on the relationship between internet usage and physical activity. In Model 3, we included age (internet_age_i) as another moderating variable to investigate its potential influence on the relationship. In Model 4, we introduced educational level (internet_edu_i) as another moderating variable to study whether education plays a role in the relationship between internet usage and physical activity. By constructing these regression models, we can comprehensively study the relationship between internet usage and residents' participation in physical activities from different perspectives, and understand whether factors such as gender, age, and educational level play a moderating role in this relationship. To ensure the robustness of the results, this study also employed the variable substitution model for analysis to validate the findings of the aforementioned models.

**Table 3:** Regression.

	**(1)**	**(2)**	**(3)**	**(4)**
	**H1**	**H2**	**H3**	**H4**
Internet	−2.707^***^	−2.709^***^	−3.088^***^	−3.073^***^
	[0.035]	[0.035]	[0.037]	[0.029]
Gender	0.045^**^	0.043^**^	0.040^**^	0.084^***^
	[0.018]	[0.018]	[0.018]	[0.018]
Age	−0.024^***^	−0.024^***^	−0.024^***^	−0.024^***^
	[0.001]	[0.001]	[0.001]	[0.001]
Edu	0.398^***^	0.403^***^	0.373^***^	0.349^***^
	[0.026]	[0.027]	[0.027]	[0.028]
Internet_gender		0.044^***^		
		[0.009]		
Internet_age			−0.572^***^	
			[0.012]	
Internet_edu				0.547^***^
				[0.010]
Income	0.019^***^	0.018^***^	0.014^***^	0.015^***^
	[0.005]	[0.005]	[0.005]	[0.005]
Urban	0.159^***^	0.157^***^	0.161^***^	0.212^***^
	[0.028]	[0.028]	[0.028]	[0.028]
Health	0.051^***^	0.052^***^	0.052^***^	0.064^***^
	[0.013]	[0.013]	[0.013]	[0.013]
_cons	0.985^***^	0.972^***^	0.976^***^	0.840^***^
	[0.151]	[0.151]	[0.150]	[0.147]
*N*	12,264	12,264	12,264	12,264

Standard errors in brackets.

^**^p < 0.05.

^***^p < 0.01.

In Model 1, we examined the impact of internet usage time (internet_ _i_) on residents' participation in physical activities (*y*_i_) to test Hypothesis 1. Through a meticulous analysis of Model 1, several findings have emerged:

Firstly, the regression coefficient of internet usage is −2.707, significant at the 1% confidence level (*p* < 0.01). This indicates that, an increase in internet usage frequency significantly reduces the frequency of physical exercise. In other words, longer internet usage time is associated with a decreased frequency of engaging in physical activities. This result confirms Hypothesis 1, suggesting that higher internet usage frequency is associated with a significant decrease in the frequency of physical activities.

Secondly, regarding control variables, gender does not significantly affect physical exercise, implying no significant difference in physical activity between males and females. The positive regression coefficient of age, significant at the 1% confidence level, suggests that individuals of older age are more likely to have a higher frequency of physical exercise when other factors are held constant. The positive and significant regression coefficient of educational level indicates that individuals with higher education tend to engage in physical exercise more frequently.

The regression coefficient for income level is positive and statistically significant at the 1% level, indicating that an increase in income significantly increases the frequency of physical exercise. The regression coefficients for both urban and rural residents are positive and statistically significant at the 1% level, with urban residents exercising more frequently than rural residents under similar conditions. Lastly, the positive and significant regression coefficient of health status indicates that individuals with better health are more likely to engage in physical exercise frequently when other conditions are held constant.

In summary, the results of Model 1 support Hypothesis 1, confirming a positive correlation between internet usage time and participation in physical activities. Factors such as age, educational level, income level, urban/rural status, and health status play different roles in this relationship. Specifically, age, educational level, and health status have significant effects on physical exercise frequency.

In Model 2, we explored the impact of the interaction between internet usage and gender on residents' participation in physical activities. The results show that the regression coefficient of the interaction term is 0.044 (*p* < 0.01), statistically significant at the 1% level. This implies that gender significantly moderates the association between internet usage and physical activity frequency. Therefore, Hypothesis 2 is also supported, indicating that gender plays a significant moderating role in the relationship between internet usage and physical activity.

In Model 3, we studied the influence of the interaction between internet usage and age on residents' participation in physical activities. The regression analysis shows that the regression coefficient of the interaction term is −0.572, significant at the 1% confidence level (*p* < 0.01). This suggests that there are significant differences in the impact of internet usage on physical exercise frequency across different age groups. Thus, Hypothesis 3 is confirmed, indicating that age plays a significant moderating role in the relationship between internet usage and physical activity.

In Model 4, we examined the influence of the interaction between internet usage and educational level on residents' participation in physical activities. However, the results of the regression analysis show that the regression coefficient of the interaction term is 0.547, significant at the 1% confidence level (*p* < 0.01). This indicates that different educational levels significantly moderate the relationship between internet usage and physical exercise frequency. Hence, Hypothesis 4 is supported, suggesting that educational level not plays a significant moderating role in the relationship between internet usage and physical activity.

In conclusion, the results of Model 2 that gender significantly moderates the relationship between internet usage and physical activity; the finding of Model 3 confirms that age plays a significant moderating role the association between internet usage and physical activity; and the outcomes of Model 4 indicated that education level significantly moderates the relationship between internet usage and physical activity.

### Robustness test

In this section, we employed a variable substitution model to validate the robustness of the previous results, as shown in Table 4. Exercise time was measured by coding the frequency of physical exercise, with nearly daily exercise coded as 1 and other frequencies as 0, serving as the dependent variable in measuring physical activity. Estimations were conducted using a binary Probit model. The coefficients for the core explanatory variable, internet usage, were negative and statistically significant, mirroring the baseline regression results, indicating the empirical robustness of the earlier findings. In Models 6–7, the significance of the interaction terms, consistent with the previous data regression results in Table 3. There was a slight variation in the significance of the educational interaction term in Model 8, still consistent with the previous data regression results in Table 3. Consequently, we can conclude that Hypotheses 1, 2, 3, and 4 of this study have all been further verified.

**Table 4:** Robustness test.

	**(5)**	**(6)**	**(7)**	**(8)**
	**H1**	**H2**	**H3**	**H4**
Internet	−2.441^***^	−2.438^***^	−2.850^***^	−2.922^***^
	[0.374]	[0.378]	[0.384]	[0.322]
Gender	0.057^***^	0.056^***^	0.051^**^	0.090^***^
	[0.021]	[0.021]	[0.021]	[0.020]
Age	−0.013	−0.013	−0.016^*^	−0.017^*^
	[0.009]	[0.009]	[0.009]	[0.009]
Edu	0.402^***^	0.406^***^	0.380^***^	0.361^***^
	[0.018]	[0.018]	[0.020]	[0.029]
Internet_gender		0.044^***^		
		[0.010]		
Internet_age			−0.510^***^	
			[0.086]	
Internet_edu				0.509^***^
				[0.069]
Income	0.020^***^	0.020^***^	0.016^***^	0.016^***^
	[0.006]	[0.006]	[0.005]	[0.005]
Urban	0.279^***^	0.278^***^	0.262^***^	0.285^***^
	[0.092]	[0.092]	[0.096]	[0.100]
Health	0.089^**^	0.090^***^	0.084^**^	0.087^**^
	[0.035]	[0.034]	[0.036]	[0.037]
_cons	−0.279	−0.307	−0.074	0.083
	[1.052]	[1.053]	[1.049]	[1.079]
*N*	12,264	12,264	12,264	12,264

Standard errors in brackets.

^*^p < 0.1.

^**^p < 0.05.

^***^p < 0.01.

## Conclusion and discussion

Previous studies have investigated various factors related to physical activity participation, such as social online network (46) and social class (17). However, the specific relationship between internet usage and physical activity in the Chinese context has not been well understood. This study addresses this gap by utilizing data from the 2017 China General Social Survey (CGSS) to examine the impact of internet usage on the physical activity participation of Chinese residents. Additionally, we analyze the role of different individual characteristics in shaping this association.

Firstly, regarding the negative association between internet usage and physical activity participation, this outcome may stem from the role of the internet as an information acquisition and social tool (47). With the proliferation of the internet, individuals can more easily access knowledge about health and physical exercise, enabling them to find suitable ways to engage in various physical activities (48). Online social platforms also provide opportunities and easily access for communication and sharing of exercise experiences, thereby fostering interest in physical activity. This observed negative correlation may be particularly relevant to the characteristics of the study sample, which consists predominantly of middle-aged and older adults (49). The validation of this association suggests that the impact of internet usage on the frequency of physical exercise is substantial within this demographic group. Notably, older individuals, who may reduce their dependence on the internet, allocate their leisure time to engage in physical activities. This demographic nuance may contribute to the observed negative relationship between internet usage and physical exercise frequency.

Secondly, gender and educational level play a moderating role in the relationship between internet usage and physical activity. This may reflect that in the internet era, gender differences persist in physical exercise, consistent with previous research findings (50). Due to societal roles and expectations, males are more likely to engage in physical exercise, while females may face certain constraints. Despite the increase in female participation in physical activities and the narrowing of the gender gap with changing social norms and the prevalence of the internet, gender still moderates the relationship between internet usage and physical activity. Additionally, the impact of educational level on the relationship may be attributed to the varying accessibility and utilization of information through the internet based on different educational backgrounds.

Finally, the moderating role of age in the relationship between internet usage and physical activity may reflect the unique needs of middle-aged and older adults in terms of physical activity. As people age, their focus on health and the demand for physical activity gradually increase. With the help of the internet, middle-aged and older adults can access more exercise advice and guidance tailored to their age and health conditions, thus actively participating in physical activities (51). This trend may further enhance the moderating effect of age on the relationship between internet usage and physical activity. For instance, they are more likely to discover exercise routines from peers of the same age on platforms such as TikTok or WeChat. Governments and health organizations can leverage these findings to utilize internet platforms for providing scientific knowledge, fitness guidance, and social support related to physical exercise. Tailoring health education for different age groups, genders, and educational levels can enhance physical activities participation and overall public health.

The conclusions of this study can be explained from the perspective of the internet as a tool for information acquisition and social interaction. Additionally, they reflect the shifting social norms and the specific health needs of middle-aged and older adults. However, the study has several limitations. First, although the 2017 China General Social Survey (CGSS) is the most comprehensive survey data available on physical activities participation in China to date, factors such as the emergence of the COVID-19 pandemic and the proposal of national sports policies and guidelines in recent years are bound to have an impact on residents' physical activity participation. The cross-sectional data used in this paper may not capture long-term trends and causal relationships, limiting the comprehensive understanding of the relationship between internet usage and physical activity. Second, the omission of other potential influencing factors such as cultural and geographical differences might overlook important determinants. Future research could employ longitudinal data and consider more potential factors to provide a more accurate depiction of the relationship between internet usage and physical activity. Third, when conducting social surveys, CGSS could not include residents' participation motivation in physical activities. Future research should focus on follow-up surveys on participation in physical activities.

Based on the above discussions, this study provides the following insights for promoting resident participation in physical activities. First, China should increase the Internet access rate of residents, popularize Internet use knowledge, and enable more residents to join the Internet. At the same time, China should also provide guidance on the use of the internet, allowing residents to participate in physical activities on the premise of healthy internet use. Second, the government and social organizations should guide and encourage residents to have access to physical activities and fitness related knowledge during the use of the internet. Third, China should promote the combination of the Internet and physical activities, and provide convenience for residents to use the Internet to participate in physical activities. Lastly, the Chinese government should formulate differentiated policies for different groups based on age, gender and education differences, and create conditions for different groups to use the Internet and participate in physical activities.

## Data availability statement

The original contributions presented in the study are included in the article/supplementary material, further inquiries can be directed to the corresponding author.

## Author contributions

MJ: Writing – original draft, Writing – review & editing, Conceptualization, Data curation, Formal analysis, Methodology, Project administration, Resources, Software, Supervision. DD: Conceptualization, Formal analysis, Investigation, Methodology, Project administration, Writing – original draft, Writing – review & editing. XY: Writing – review & editing, Writing – original draft, Data curation, Funding acquisition, Methodology.
